# Bioassay-Guided Isolated Compounds from *Morinda officinalis* Inhibit Alzheimer’s Disease Pathologies

**DOI:** 10.3390/molecules22101638

**Published:** 2017-09-29

**Authors:** Yoon Kyoung Lee, Hyo Jeong Bang, Jeong Bin Oh, Wan Kyunn Whang

**Affiliations:** Pharmaceutical Botany Laboratory, College of Pharmacy, Chung-Ang University, Heukseok-dong, Dongjak-gu, Seoul 151-756, Korea; dbsrudaks486@naver.com (Y.K.L.); bhj1027@hanmi.co.kr (H.J.B.); ojb6911@naver.com (J.B.O.)

**Keywords:** *Morinda officinalis*, bioassay-guided isolation, Anthraquinone, Alzheimer’s diseases, structure-activity relationship

## Abstract

Due to the side effects of synthetic drugs, the therapeutic potential of natural products for Alzheimer’s disease (AD) has gained interest. *Morinda officinalis* has demonstrated inhibitory effects on geriatric diseases, such as bone loss and osteoporosis. However, although AD is a geriatric disease, *M. officinalis* has not been evaluated in an AD bioassay. Therefore, *M. officinalis* extracts and fractions were tested for AD-related activity, including inhibition of acetylcholinesterase (AChE), butyrylcholinesterase (BChE), β-site amyloid precursor protein cleaving enzyme 1 (BACE1), and advanced glycation end-product (AGE) formation. A bioassay-guided approach led to isolation of 10 active compounds, eight anthraquinones (**1**–**8**), one coumarin (**9**), and one phytosterol (**10**), from n-hexane and ethyl acetate fractions of *M. officinalis*. The five anthraquinones (**4**–**8**) were stronger inhibitors of AChE than were other compounds. Compounds **3** and **9** were good inhibitors of BChE, and compounds **3** and **8** were good inhibitors of BACE1. Compounds **1**–**5** and **7**–**9** were more active than the positive control in inhibiting AGE formation. In addition, we first suggested a structure-activity relationship by which anthraquinones inhibit AChE and BACE1. Our findings demonstrate the preventive and therapeutic efficacy of *M. officinalis* for AD and its potential use as a natural alternative medicine.

## 1. Introduction

*Morinda officinalis* How. is a member of the Rubiaceae family and grows widely in subtropical and tropical climates [1]. *M. officinalis* is distributed in Southern China and Northeast Asia and is used to treat sexual impotence, spermatorrhea, irregular menstruation, menstrual disorders, osteoporosis, diabetes mellitus, and inflammatory diseases such as rheumatoid arthritis and dermatitis [2,3]. Moreover, several studies have reported that *M. officinalis* has various biological activities, including protecting against bone loss [4], osteoporosis [5,6], age-induced bone degeneration [7], and has anti-oxidant [8], anti-fatigue [9], and anti-inflammatory actvities [10]. The compounds isolated from *M. officinalis* include polysaccharides, flavone glycosides, iridoid glycosides, anthraquinones, coumarins, and phytosterols, such as rubiadin, rubiadin-1-methyl ether, 2-hydroxy-1-methoxy-anthraquinone, 1,3,8-trihydroxy-2-methoxy-anthraquinone, morindolide, morofficinaloside, asperuloside, asperulosidic acid, monotropein, scopoletin, stigmasterol, daucosterol, and β-sitosterol [3,11,12].

Alzheimer’s disease (AD) is major form of dementia and one of the most common age-related progressive and irreversible neurodegenerative diseases. It is accompanied by memory loss, cognitive dysfunction, disorientation, behavioral disturbances, and personality changes [13,14,15]. The two most common hypotheses that characterize AD pathology are the cholinergic and amyloid hypotheses [16]. According to the cholinergic hypothesis, AD is caused by a deficiency of the neurotransmitter acetylcholine, which is hydrolyzed by acetylcholinesterase (AChE) and butyrylcholinesterase (BChE) [17,18]. Therefore, cholinesterases, including AChE and BChE, are key enzymes in AD pathogenesis [19,20]. The amyloid hypothesis suggests that amyloid-β peptide (Aβ) accumulation in the brain is critical in AD pathogenesis [21,22]. Aβ is formed from sequential proteolytic cleavage of amyloid precursor protein (APP) by the aspartic protease γ- and β-secretase (BACE1) in the amyloidogenic pathway [23,24,25]. APP cleavage by BACE1 increases the production and accumulation of neurotoxic forms of Aβ in the brain and causes neurodegeneration [26,27]. In addition, a previous study reported that advanced glycation end-products (AGEs) contribute to neuronal dysfunction and death in the progression of various neurodegenerative diseases including AD [28]. Accordingly, inhibiting cholinesterases, AGE formation, and Aβ accumulation are important in preventing AD.

To treat AD, synthetic drugs, such as tacrine, rivastigmine, donepezil, and galantamine, are usually prescribed. However, these drugs have side effects (e.g., hepatotoxic gastrointestinal disturbances) and problems with bioavailability [29,30,31]. Due to these side effects, the therapeutic potential of natural products has received great interest. Although studies have assessed the activity of anthraquinones on AD [27], the effects of *M. officinalis*, which contains anthraquinones, on AD have not been evaluated. Therefore, we isolated major components from *M. officinalis* and tested their inhibitory activities on AChE, BChE, BACE1, and AGE formation.

## 2. Results

### 2.1. Identification of Compounds ***1**–**10*** Isolated from M. officinalis

**Figure 1:**
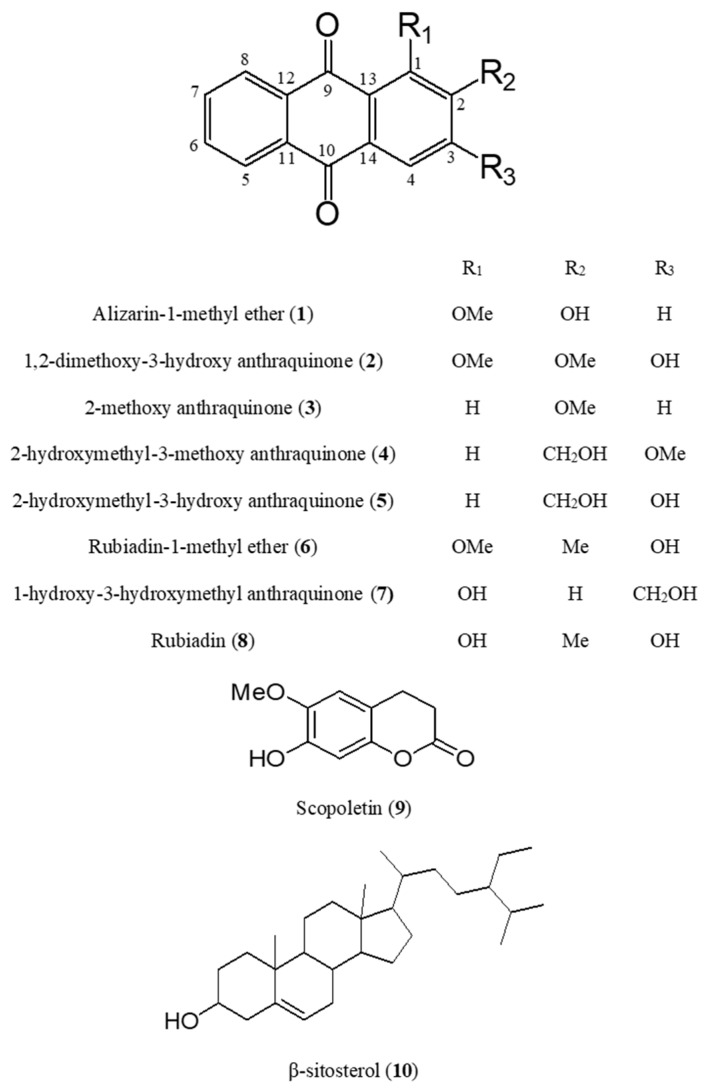
Structures of compounds **1**–**10**.

**Figure 2:**
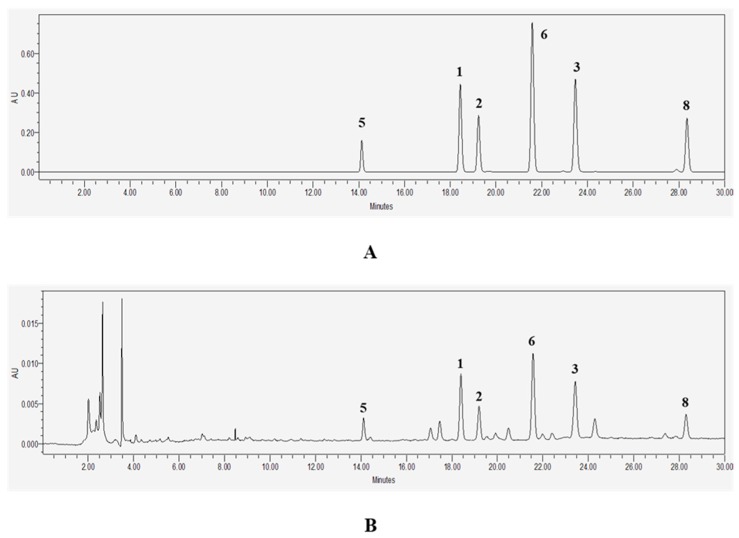
Chromatograms of standards mixture (**A**) and *M. officinalis* crude MeOH extract (**B**).

According to the bioassay-guided isolation method, we chromatographically separated the *M. officinalis* Hx and EA fractions. As a result, eight anthraquinones (**1**–**8**), one coumarin (**9**), and one phytosterol (**10**) were isolated. Compounds **1**–**10** isolated from *M. officinalis* were identified as alizarin-1-methyl ether (**1**), 1,2-dimethoxy-3-hydroxy anthraquinone (**2**), 2-methoxy anthraquinone (**3**), 2-hydroxymethyl-3-methoxy anthraquinone (**4**), 2-hydroxymethyl-3-hydroxy anthraquinone (**5**), rubiadin-1-methyl ether (**6**), 1-hydroxy-3-hydroxymethyl anthraquinone (**7**), rubiadin (**8**), scopoletin (**9**), and β-sitosterol (**10**) [3,5,12,32,33] by comparison with spectroscopic (^1^H-, ^13^C-NMR) and LC-MS data from the literature (Figure 1). The *m*/*z* data and retention time of each compound were provided in Table 1. Observed mass value accuracies of compounds **1**–**10** were credible to 5 ppm. After identifying compounds **1**–**10**, HPLC analysis was conducted to determine the major components of *M. officinalis* extracts (Figure 2).

### 2.2. AChE, BChE, BACE1, and AGE Formation Inhibitory Activities of the Extracts and Fractions from M. officinalis 

To demonstrate the potential of *M. officinalis* to prevent AD, we examined the effects of *M. officinalis* root extracts and fractions on AChE, BChE, BACE1, and AGE formation. The results are summarized in Table 2. The IC_50_ values of positive control in AChE, BChE, BACE1, and AGEs formations were judged suitable compared with previous literatures [16,26,27,34]. The *M. officinalis* extracts, Hx, and EA fractions significantly inhibited AChE activity (IC_50_ of 58.82 ± 9.13, 33.66 ± 4.73, and 80.14 ± 16.65 μg/mL, respectively). Although *M. officinalis* extracts slightly inhibited BChE activity, the Hx fraction showed the highest inhibition with an IC_50_ of 105.99 ± 0.69 µg/mL. The extracts, Hx, and EA fractions were the most potent BACE1 inhibitors with IC_50_ values of 24.40 ± 2.84, 42.36 ± 3.94, and 64.45 ± 4.22 µg/mL, respectively. Finally, the Hx fraction (IC_50_ of 166.03 ± 7.76 µg/mL) most strongly inhibited AGE formation, followed by the EA fraction (IC_50_ of 417.92 ± 14.29 µg/mL), and the extracts had no activity.

### 2.3. AChE, BChE, BACE1, and AGE Formation Inhibitory Activities of Compounds ***1**–**10*** Isolated from M. officinalis

Compounds **1**–**10** were tested for their ability to inhibit AChE, BChE, BACE1, and AGE formation. The results were shown in Table 3. The IC_50_ values of positive control in AChE, BChE, BACE1, and AGEs formations were also judged suitable compared with the previous literature [16,26,27,35]. β-sitosterol (**10**) did not inhibit any of the tested activities with IC_50_ values > 500 μM or ND (not detected). Five anthraquinones (**4**–**8**) were stronger AChE inhibitors than were the other compounds. The IC_50_ values of compounds **4**–**8** were 27.05 ± 1.49, 19.06 ± 3.58, 87.19 ± 6.56, 96.38 ± 17.23, and 44.31 ± 12.20 μM, respectively. Compounds **3** and **9**, had mild activity toward AChE and inhibited AChE more significantly than did the other compounds with IC_50_ values of 230.18 ± 5.97 and 50.43 ± 1.61 μM, respectively. Furthermore, compounds **3** (IC_50_ of 9.29 ± 1.92 μM) and **8** (IC_50_ of 19.82 ± 3.05 μM) showed greater BACE1 inhibition than did quercetin (IC_50_ of 22.75 ± 1.20 μM), the positive control. Compound **6** had activity similar to the positive control with an IC_50_ of 25.89 ± 2.11 μM. Compounds **1**–**5** and **7**–**9** inhibited AGE formation more than AG, the positive control. Compound **9** was the best inhibitor of AGE formation with an IC_50_ of 5.43 ± 0.11 μM.

## 3. Discussion

In recent years, the aging society and increasing life span have increased the number of people over 65 years old worldwide. As a result, degenerative and geriatric diseases are increasing. Dementia, a major symptom of cognitive disorders, is a significant social problem [36]. While dementia can result from degenerative dementia, senile dementia, Parkinson's disease, and AD, AD is the most common, accounting for 50% to 60% of all dementia [37]. *M. officinalis* has already been demonstrated to inhibit geriatric diseases such as bone loss and osteoporosis. Although AD is a geriatric disease, *M. officinalis* has not been evaluated in an AD bioassay. Therefore, we aimed to assess whether *M. officinalis* has the potential to treat AD by inhibit AChE, BChE, BACE1, and AGE formation.

*M. officinalis* extracts and fractions were investigated for their ability to inhibit AChE, BChE, BACE1, and AGE formation. The *M. officinalis* extracts were good inhibitors of AChE, BChE, and BACE1. The extracts inhibited BACE1 more strongly than did the other fractions. The Hx fraction was a stronger inhibitor in all assays. The Hx fraction inhibited AChE, BChE, and AGE formation significantly more than the other fractions. The EA fraction mildly inhibited AChE, BACE1, and AGE formation. In contrast, the BuOH and water fractions had no, or slight, activity in all assays. These results demonstrated that the potential of *M. officinalis* extracts to prevent AD was derived from the Hx and EA fractions.

Therefore, we conducted bioassay-guided isolation from the Hx and EA fractions. We isolated bioactive compounds, including eight anthraquinones (**1**–**8**), one coumarin (**9**), and one phytosterol (**10**). The isolated compounds **1**–**10** were investigated for inhibition of AChE, BChE, BACE1, and AGE formation. Previous literatures studied AD activities of various natural products, for examples, cholinesterase activities of flavonoid isolated from *Kaempferia parviflora*, *Maclura pomifera*, essential oils of *Salvia* species, and their crude extracts [35,38,39]. When we compared previous articles with our data, it could know that anthraquinones had more potential than the natural products kind of flavonoids and fatty acids. Taken together, our study was significant to have accessed the anti-AD activities of anthraquinones.

Compounds **4**–**8** were stronger AChE inhibitors than other compounds. Of these, compound **5** was the most active. Furthermore, we uncovered the following relationships between the anthraquinone structure and AChE inhibitory activity: (1) anthraquinones with no substituent on C-1 (compounds **4** and **5**) were more active than those with a substituent in C-1 (compounds **1**–**3** and **6**–**8**); (2) anthraquinone with a substituted methyl group on C-2 (compounds **6** and **8**) were more active than those with a methoxy group (compounds **2** and **3**); (3) anthraquinones with a substituent on C-3 (compounds **2** and **4**–**8**) had stronger activity than those without (compounds **1** and **3**); (4) anthraquinones with a hydroxy group at C-3 (compounds **5** and **8**) were more active than those with a methoxy group (compounds **4** and **6**); and (5) the anthraquinone with no hydroxy group was a minor inhibitor (compound **3**).

Compound **9** significantly inhibited BChE, and compound **3** slightly inhibited AChE, making them the most active among the isolated anthraquinones. According to bioassay-guided isolation, the EA fraction also showed low potential, because most anthraquinones isolated from the EA fraction had weak activity. The Hx fraction was the most active because compound **3**, a good inhibitor, was isolated from Hx fraction.

Compounds **3** and **8** were stronger BACE1 inhibitors than quercetin, a positive control. Compound **6** showed similar activity to the positive control. Compound **3** was the best BACE1 inhibitor. We suggested the following structure-activity relationship for BACE1 inhibition by anthraquinones: (1) anthraquinones with only one substituent (compound **3**) were more active than those with more substituents (compounds **1**, **2**, and **4**–**8**); (2) anthraquinones with all substituents on C-1, 2, or 3 (compounds **2**, **6**, and **8**) were more active than those with two substituents (compounds **1**, **4**, **5**, and **7**); (3) hydroxy (compound **8**), methyl (compound **6**), and methoxy group (compound **2**) substituents had the highest activity in that order; and (4) when anthraquinones have two substituents, the substituent position determines the activity. C-1 and 3 (compound **7**), C-1 and 2 (compound **1**), and C-2 and 3 (compounds **4** and **5**) were the most active in that order.

Finally, compounds **1**–**5**, **7**, and **8** were stronger inhibitors of AGE formation than AG, the positive control. Compound **9** showed the best activity. Previous studies have indicated that scopoletin (**9**) is a remarkable inhibitor of AGE formation [40]. Our results indicated that anthraquinones with only one substituent (compound **3**) were the most effective, anthraquinones with a hydroxy group (compounds **5** and **8**) had more activity than those with other substituents (compounds **4** and **6**), and anthraquinones with a methoxy group (compound **2**) were stronger inhibitors than those with a methyl group (compound **6**).

In conclusion, this study used bioassay-guided isolation to identify 10 compounds from *M. officinalis*. The isolated compounds inhibited AChE, BChE, BACE1, and AGE formation, which are related to AD. In addition, we suggested a structure-activity relationship for AChE and BACE1 inhibition by anthraquinones. These results demonstrated that *M. officinalis* root extracts were therapeutic and may be a natural medicine for treating AD.

## 4. Materials and Methods

### 4.1. Plant Materials

*M. officinalis* roots were purchased from Kyung-Dong market, Seoul, Korea. Prof. Whang Wan Kyunn identified the *M. officinalis*.

### 4.2. Instruments and Reagents

*n*-Hexane (Hx), ethyl acetate (EA), *n*-butanol (BuOH), methanol (MeOH), ethanol (EtOH), and distilled water were used for extraction, fractionation, and open column chromatography. Open column chromatography used Sephadex LH-20 (25–100 μm; Pharmacia, Stockholm, Sweden), MCI CHP 20P (Supelco, St. Louis, MO, USA), and ODS gel (400–500 mesh; Waters, Milford, MA, USA). Dimethyl sulfoxide-*d*_6_ (DMSO-*d*_6_) and chloroform-*d* (CDCl_3_) were used for the NMR solution. MS was performed with ultra-high performance liquid chromatography and high-resolution mass spectrometry (UHPLC-HRMS) coupled with electrospray ionization hybrid linear trap-quadruple-Orbitrap MS (ESI/LTQ-Orbitrap) on an Ultimate 3000 rapid separation liquid chromatography (RSLC) system (Thermo, Darmstadt, Germany). ^1^H- and ^13^C-nuclear magnetic resonance (NMR) spectra were collected at 600 and 150 MHz, respectively, with a JEOL spectrometer. Chemical shifts are expressed as parts per million (ppm) on the δ scale, and coupling constants (*J*) are shown in Hertz. HPLC was conducted with Empower Pro 2.0 software (Waters, Milford, MA, USA), and determination was performed with a Waters 2695 system pump and Waters 996 Photodiode array detector (Waters, Milford, MA, USA). The separation column was a Waters Sunfire™ C18 column (4.6 × 250 mm, 5 μm). HPLC-grade solvents, such as acetonitrile (ACN), methanol (MeOH), and distilled water (H_2_O), were purchased from J. T. Baker^®^ (Phillipsburg, PA, USA). HPLC-grade phosphoric acid and dimethyl sulfoxide (DMSO) were obtained from DEAJUNG Chemical (Siheung, Gyeonggi, Korea). Reagents and solvents including electric eel AChE (EC3.1.1.7), horse serum BChE (EC3.1.1.8), acetylthiocholine iodide (ACh), butyrylthiocholine chloride (BCh), 5,5′-dithiobis [2-nitrobenzoic acid] (DTNB), berberine, bovine serum albumin, aminoguanidine (AG), glucose, and fructose were purchased from Sigma-Aldrich Chemical Company (St. Louis, MO, USA). The BACE1 FRET assay kit (β-secretase) was purchased from PanVera Co. (Madison, WI, USA).

### 4.3. Extraction, Fractionation, and Isolation of M. officinalis 

Dried and powdered *M. officinalis* roots (3.9 kg) were extracted in MeOH (20 L × 3) at room temperature. The filtrate was concentrated to dryness (613.4 g) in vacuo; suspended in water (H_2_O); and partitioned in Hx, EA, and BuOH depending on solvent polarity. The result yielded Hx (3.84 g), EA (7.23 g), BuOH (192.81 g), and water (270.42 g) fractions. Among these three fractions, the Hx and EA fractions showed the most potent activities in the four anti-AD model assays. Therefore, we executed isolation from Hx and EA fractions.

The Hx fraction was subjected to Sephadex LH-20 chromatography and eluted in increasing MeOH:water (60:40 to 100:0) solutions yielding eight sub-fractions. Sub-fraction 3 was separated on a Sephadex LH-20 column (Pharmacia, Stockholm, Sweden) with 50% MeOH to obtain fractions 3-1 to 3-3. Sub-fraction 3-2 was separation on an MCI gel with 80% MeOH to yield four fractions. Sub-fractions 3-2-2 and 3-2-3 were separated on an ODS column and eluted with 60% MeOH. Fraction 3-2-2-2 was separated on Sephadex LH-20 with 50% MeOH to isolate compound **1**. Sub-fraction 3-2-3-3 was separated on Sephadex LH-20 with 40% MeOH, and sub-fraction 3-2-3-3-3 was separated on ODS (50% MeOH) to yield compound **2**. Compound **3** was isolated from fraction 5-2.

A portion of the EA fraction was separated on a Sephadex LH-20 column with an elution gradient of 60% to 100% MeOH to give nine sub-fractions. Sub-fraction 3 was separated on a Sephadex LH-20 column with 40% MeOH to yield sub-fractions 3-1 to 3-11. Sub-fraction 3-6 was separated by MCI column chromatography with 50% MeOH, and three fractions (3-6-1 to 3-6-3) were collected. Fraction 3-6-2 was separated by ODS eluted with 60% MeOH. Sub-fraction 3-6-2-2 was separated on Sephadex LH-20 with 50% MeOH leading to the isolation of compounds **4** and **5**. Fraction 3-7 was separated by MCI eluted with 80% MeOH to yield compound **6** and sub-fractions 3-7-1 to 3-7-8. Fraction 3-7-7 was applied to an ODS column with 60% MeOH, yielding compound **7**. Sub-fraction 3-10 was separated by MCI (50% MeOH), MCI (80% MeOH), and ODS (60% MeOH) yielding compound **8**. Fractions 2 and 8 were recrystallized to isolated compounds **9** and **10**, respectively.

### 4.4. Identification of Compounds Isolated from M. officinalis

#### 4.4.1. NMR

*Compound* **1**: C_15_H_10_O_4_; ESI/LTQ-Orbitrap-HRMS *m*/*z*: 255.0652 [M + H]^+^; ^1^H-NMR (600 MHz, DMSO-*d*_6_) δ: 8.05 (2H, m, H-5, 8), 7.83 (1H, d, *J* = 8.4 Hz, H-3), 7.78 (2H, m, H-6, 7), 7.17 (1H, d, *J* = 8.4 Hz, H-4), 3.78 (3H, s, 1-OMe); ^13^C-NMR (150 MHz, DMSO-*d*_6_) δ: 182.6 (C-10), 180.9 (C-9), 160.9 (C-2), 148.2 (C-1), 134.5 (C-13), 133.6 (C-6, 7), 132.7 (C-14), 126.5 (C-3), 126.4 (C-11, 12), 125.9 (C-5), 125.2 (C-8), 121.9 (C-4), 57.8 (1-OMe).

*Compound* **2**: C_15_H_10_O_3_; ESI/LTQ-Orbitrap-HRMS *m*/*z*: 285.0758 [M + H]^+^; ^1^H-NMR (600 MHz, DMSO-*d*_6_): 8.02 (1H, d, *J* = 7.2 Hz, H-8), 7.96 (1H, d, *J* = 7.2 Hz, H-5), 7.75 (1H, t, *J* = 7.8, 7.2 Hz, H-7), 7.67 (1H, t, *J* = 7.8, 7.2 Hz, H-6), 7.11 (1H, s, H-4), 3.79 (3H, s, 1-OMe), 3.72 (3H, s, 2-OMe); ^13^C-NMR (150 MHz, DMSO-*d*_6_): 184.2 (C-10), 178.6 (C-9), 155.5 (C-1, 3), 148.8 (C-2), 136.1 (C-12), 134.4 (C-7), 132.8 (C-6), 132.7 (C-11), 131.4 (C-14), 126.6 (C-8), 126.1 (C-5), 115.2 (C-4, 13), 61.1 (1-OMe), 59.9 (2-OMe).

*Compound* **3**: C_15_H_10_O_3_; ESI/LTQ-Orbitrap-HRMS *m*/*z*: 239.0706 [M + H]^+^; ^1^H-NMR (600 MHz, DMSO-*d*_6_): 8.11 (1H, d, *J* = 6.3 Hz, H-8), 8.05 (1H, d, *J* = 6.2 Hz, H-5), 7.82 (2H, m, H-6, 7), 7.49 (2H, s, H-1, 3), 7.12 (1H, s, H-4), 3.78 (3H, s, 2-OMe); ^13^C-NMR (150 MHz, DMSO-*d*_6_): 185.4 (C-10), 181.8 (C-9), 157.1 (C-2), 134.4 (C-12), 134.2 (C-7), 134.0 (C-6), 133.5 (C-11), 132.9 (C-14), 129.9 (C-1), 129.1 (C-3), 126.7 (C-8), 126.5 (C-5), 126.2 (C-13), 111.3 (C-4), 59.8 (2-OMe).

*Compound* **4**: C_16_H_12_O_4_; ESI/LTQ-Orbitrap-HRMS *m*/*z*: 267.0653 [M − H]^−^; ^1^H-NMR (600 MHz, DMSO-*d*_6_): 8.06 (1H, d, *J* = 7.8 Hz, H-8), 7.98 (1H, d, *J* = 7.2 Hz, H-5), 7.78 (1H, t, *J* = 7.8, 7.2 Hz, H-7), 7.69 (1H, t, *J* = 7.8, 7.2 Hz, H-6), 6.97 (2H, s, H-1, 4), 4.56 (2H, s, 2-CH_2_OH), 3.68 (3H, s, 3-OMe); ^13^C-NMR (150 MHz, DMSO-*d*_6_): 184.4 (C-10), 182.8 (C-9), 137.2 (C-3), 135.6 (C-14), 135.1 (C-7), 134.0 (C-6), 132.3 (C-11), 132.1 (C-12), 128.1 (C-1), 126.2 (C-5, 8), 125.5 (C-2, 13), 114.7 (C-4), 62.3 (2-CH_2_OH), 61.0 (3-OMe).

*Compound* **5**: C_15_H_10_O_4_; ESI/LTQ-Orbitrap-HRMS *m*/*z*: 253.0503 [M + H]^+^; ^1^H-NMR (600 MHz, DMSO-*d*_6_): 8.08 (1H, d, *J* = 7.2 Hz, H-8), 8.05 (1H, d, *J* = 7.2 Hz, H-5), 7.97 (1H, s, H-4), 7.79 (1H, t, *J* = 7.8, 7.2 Hz, H-7), 7.74 (1H, t, *J* = 7.8, 7.2 Hz, H-6), 7.12 (1H, s, H-1), 4.49 (2H, s, 2-CH_2_OH); ^13^C-NMR (150 MHz, DMSO-*d*_6_): 184.6 (C-10), 180.4 (C-9), 160.3 (C-3), 134.7 (C-14), 134.5 (C-7), 134.4 (C-6), 133.7 (C-11), 133.4 (C-12), 126.8 (C-1), 126.7 (C-5, 8), 126.6 (C-2, 13), 114.1 (C-4), 60.4 (2-CH_2_OH).

*Compound* **6**: C_16_H_12_O_4_; ESI/LTQ-Orbitrap-HRMS *m*/*z*: 269.0808 [M + H]^+^; ^1^H-NMR (600 MHz, DMSO-*d*_6_): 8.08 (1H, d, *J* = 6.6 Hz, H-8), 8.02 (1H, d, *J* = 6.6 Hz, H-5), 7.82 (1H, t, *J* = 6.6, 7.2 Hz, H-7), 7.76 (1H, t, *J* = 6.6, 7.2 Hz, H-6), 7.41 (1H, s, H-4), 3.72 (3H, s, 1-OMe), 2.09 (3H, s, 2-Me); ^13^C-NMR (150 MHz, DMSO-*d*_6_): 183.5 (C-10), 180.1 (C-9), 164.8 (C-1), 161.2 (C-3), 135.2 (C-7), 134.9 (C-6), 134.2 (C-12), 133.5 (C-11), 132.6 (C-14), 127.0 (C-8), 126.7 (C-5), 126.4 (C-4), 116.9 (C-13), 110.4 (C-2), 60.9 (1-OMe), 9.6 (2-Me).

*Compound* **7**: C_15_H_10_O_4_; ESI/LTQ-Orbitrap-HRMS *m*/*z*: 253.0495 [M − H]^−^; ^1^H-NMR (600 MHz, DMSO-*d*_6_): 8.13 (1H, d, *J* = 7.2 Hz, H-8), 8.06 (1H, d, *J* = 7.2 Hz, H-5), 7.83 (1H, t, *J* = 6.6, 7.2 Hz, H-7), 7.77 (1H, t, *J* = 6.6, 7.2 Hz, H-6), 6.98 (2H, s, H-2, 4), 4.35 (2H, s, 3-CH_2_OH); ^13^C-NMR (150 MHz, DMSO-*d*_6_): 183.5 (C-9), 183.2 (C-10), 177.2 (C-1), 165.2 (C-3), 134.9 (C-6), 134.6 (C-7), 134.0 (C-14), 133.9 (C-11), 133.3 (C-12), 127.1 (C-5), 126.0 (C-8), 126.5 (C-2), 116.5 (C-4), 112.5 (C-13), 57.9 (3-CH_2_OH).

*Compound* **8**: C_15_H_10_O_4_; ESI/LTQ-Orbitrap-HRMS *m*/*z*: 255.0654 [M + H]^+^; ^1^H-NMR (600 MHz, DMSO-*d*_6_): 8.14 (1H, d, *J* = 7.8 Hz, H-8), 8.07 (1H, d, *J* = 6.6 Hz, H-5), 7.84 (1H, t, *J* = 7.2 Hz, H-7), 7.80 (1H, t, *J* = 7.2 Hz, H-6), 7.11 (1H, s, H-4), 1.99 (3H, s, 2-Me); ^13^C-NMR (150 MHz, DMSO-*d*_6_): 185.1 (C-10), 183.0 (C-9), 165.6 (C-1), 163.1 (C-3), 134.9 (C-7), 134.3 (C-6), 134.1 (C-12), 133.4 (C-11), 132.2 (C-14), 127.0 (C-8), 126.6 (C-5), 117.4 (C-4), 110.0 (C-13), 107.9 (C-2), 8.7 (2-Me).

*Compound* **9**: C_10_H_8_O_4_; ESI/LTQ-Orbitrap-HRMS *m*/*z*: 193.0497 [M + H]^+^; ^1^H-NMR (600 MHz, DMSO-*d*_6_) δ: 7.83 (1H, d, *J* = 9.0 Hz, H-4), 7.14 (1H, s, H-5), 6.72 (1H, s, H-8), 6.16 (1H, d, *J* = 9.6 Hz, H-3), 3.76 (3H, s, 6-OMe); ^13^C-NMR (150 MHz, DMSO-*d*_6_) δ: 161.2 (C-2), 151.6 (C-7), 150.0 (C-9), 145.7 (C-6), 144.9 (C-4), 112.1 (C-3), 111.0 (C-10), 110.0 (C-5), 103.2 (C-8), 56.4 (6-OMe).

*Compound* **10**: C_29_H_50_O; ESI/LTQ-Orbitrap-HRMS *m*/*z*: 437.3768 [M + Na]^+^; ^1^H-NMR (600 MHz, CDCl_3_) δ: 5.35 (1H, m, H-6), 3.51 (1H, m, H-3), 1.99 (2H, m, H-11) 1.01 (3H, s, H-19), 0.93 (3H, m, H-21), 0.86 (3H, m, H-27), 0.83 (3H, m, H-26), 0.81 (3H, m, H-29), 0.68 (3H, s, H-18); ^13^C-NMR (150 MHz, CDCl_3_) δ: 140.8 (C-5), 121.7 (C-6), 71.8 (C-3), 56.9 (C-14), 56.0 (C-17), 50.1 (C-9), 45.8 (C-24), 42.3 (C-13), 40.4 (C-12), 39.8 (C-4), 37.3 (C-1), 36.5 (C-10), 36.1 (C-20), 33.9 (C-22), 31.9 (C-7, 8), 31.7 (C-2), 29.1 (C-25), 28.2 (C-16), 26.1 (C-23), 24.3 (C-15), 23.1 (C-28), 21.2 (C-11), 19.8 (C-26), 19.4 (C-19), 19.1 (C-27), 19.0 (C-21), 12.2 (C-29), 12.0 (C-18).

#### 4.4.2. UHPLC-ESI/LTQ-Orbitrap-HRMS Conditions

Molecular weights of the isolated compounds were confirmed by UHPLC-ESI/LTQ-Orbitrap-HRMS. Samples were dissolved in MeOH. The column (Hypersil GOLD C18, 2.1 × 50 mm, 1.9 μm, Thermo) and sampler temperatures were 30 °C and 15 °C, respectively. UV was not used. The mobile phase was 0.1% formic acid in water (solvent A) and 0.1% formic acid in acetonitrile (solvent B). The flow rate was 0.3 mL/min. The gradient conditions were 0–18 min, 0–50% B; 18–20 min, 50–100% B. The injection volume was 5.0 µL for the standard solution. The optimal analysis conditions were as follows: heater temperature, 300 °C; capillary temperature, 360 °C; auxiliary gas flow rate, 10 L/h; sheath gas flow rate, 45 L/h; S-lens RF level, 50.0 V; spray capillary voltage, 3.0 kV; full MS resolution, 35,000 (FWHM @ *m*/*z* 200); full MS AGC target, 3e^6^; and full MS maximum IT, 200 ms.

### 4.5. HPLC Analysis 

To analyze the major compounds from *M. officinalis*, a Waters Sunfire™ C18 column (4.6 × 250 mm, 5 μm) was used. Solvents A (0.2% formic acid in water) and B (acetonitrile) were used in linear gradients as the mobile phase (0–5 min, 15–30% B; 5–10 min, 30–40% B; 10–25 min, 40–60% B; 18–30 min, 60–80% B) at a flow rate of 1 mL/min. All eluents were filtered with a 0.45 μm PVDF syringe filter. The injection volume was 10 μL, and compounds were detected at a wavelength of 280 nm.

### 4.6. Bioactivities Assay

#### 4.6.1. Measurement of ChE Inhibitory Activities

ChE activity was detected by AChE- or BChE-mediated hydrolysis of DTNB for 15 min to form thiocholine and the yellow 5-thio-2-nitrobenzoate anion. The result was quantified by measuring the absorbance 412 nm. The assay mixture contained 0.1 M potassium phosphate buffer (pH 7.8), 0.3 U/mL AChE or BChE, 0.5 mM DTNB, 0.6 mM ACh or BCh, and the sample for a total volume of 0.2 mL. All tested samples were dissolved in 10% DMSO at five different final concentrations (10–500 µg/mL for extracts and fractions or 10–500 µM for isolated constituents). The reaction was performed in a 96-well plate. Berberine, a typical ChE inhibitor, was used as a positive control [16]. Inhibitory activity was calculated with the following formula: (Ac − As/Ac) × 100, where Ac is the change in absorbance for the control after 15 min and As is the change in absorbance for the sample after 15 min.

#### 4.6.2. Measurement of BACE1 Inhibition 

BACE1 inhibition was measured with a commercially available spectrophotometric method according to the manufacturer’s recommended protocol. The assay mixture contained 50 mM sodium acetate buffer (pH 4.5), 1.0 U/mL BACE1, substrate (750 nM Rh-EVNLDAEFK-Quencher in 50 mM ammonium bicarbonate), and sample. All tested samples were dissolved in 10% DMSO at five different final concentrations (2.5–1250 µg/mL for extracts and fractions or 2.5–1250 µM for isolated constituents). The reaction was incubated for 60 min at room temperature in the dark. BACE1 activity was determined by measuring the proteolysis of two fluorophores (Rh-EVNLDAEFK-Quencher) to form a fluorescent donor (Rh-EVNL) with an excitation of 545 nm and emission of 585 nm in a black 96-well plate. Quercetin, a typical BACE1 inhibitor, was used as a positive control [16,26,27]. Inhibition was calculated with the following formula: (Ac − As/Ac) × 100, where Ac is the change in fluorescence for the control after 60 min, and As is the change in fluorescence for the sample after 60 min.

#### 4.6.3. Measurement of Inhibition of AGE Formation

Inhibition of AGE formation was measured with a spectrophotometric method developed previously [34]. All tested samples were dissolved in 10% DMSO at five different final concentrations (10–500 µg/mL for extracts and fractions or 10–500 µM for isolated constituents). The assay mixture contained bovine serum albumin (10 mg/mL), 50 mM phosphate buffer (pH 7.4) with 0.02% sodium azide, and 0.4 M fructose and glucose. The reaction was incubated at 60 °C for 2 days. After incubating, fluorescence was measured at an excitation wavelength of 350 nm and emission of 450 nm in a black 96-well plate. Aminoguanidine (AG), a typical inhibitor of AGE formation, was used as a positive control. The inhibitory activity was calculated with the following formula: (Ac − As/Ac) × 100, where Ac is the fluorescence of the control, and As is the fluorescence of the sample.

### 4.7. Statistical Analysis

All assays were performed in triplicate. Data are presented as the mean ± standard deviation (SD) and were analyzed by one-way ANOVA. Data were considered statistically significant at *p* < 0.05.

## Figures and Tables

**Table 1:** Identification of compounds **1**–**10** in *M. officinalis* by UHPLC-ESI/LTQ-Orbitrap-HRMS analysis.

No.	Compound	Rt (min)	Formula	Mass Mode	Theoretical Mass	Observed Mass	Mass Error (Da)	Mass Accuracy (ppm)
**1**	Alizarin-1-methyl ether	7.84	C_15_H_10_O_4_	Positive	255.0652	255.0652	0.0000	0.0
**2**	1,2-dimethoxy-3-hydroxy anthraquinone	7.95	C_16_H_12_O_5_	Positive	285.0757	285.0758	0.0001	0.4
**3**	2-methoxy anthraquinone	8.61	C_15_H_10_O_3_	Positive	239.0703	239.0706	0.0003	1.3
**4**	2-hydroxymethyl-3-methoxy anthraquinone	7.15	C_16_H_12_O_4_	Negative	267.0653	267.0655	0.0002	0.7
**5**	2-hydroxymethyl-3-hydroxy anthraquinone	7.16	C_15_H_10_O_4_	Positive	253.0573	253.0574	0.0001	0.4
**6**	Rubiadin-1-methyl ether	8.29	C_15_H_10_O_4_	Positive	269.0808	269.0808	0.0000	0.0
**7**	1-hydroxy-3-hydroxymethyl anthraquinone	8.70	C_16_H_12_O_4_	Negative	253.0452	253.0455	0.0003	1.2
**8**	Rubiadin	9.26	C_15_H_10_O_4_	Positive	255.0652	255.0654	0.0002	0.8
**9**	Scopoletin	5.65	C_10_H_8_O_4_	Positive	193.0495	193.0497	0.0002	1.0
**10**	β-sitosterol	13.42	C_29_H_50_O	Positive	437.3754	437.3768	0.0014	3.2

**Table 2:** IC_50_ of the *M. officinalis* extracts and fractions for acetylcholinesterase (AChE), butyrylcholinesterase (BChE), β-site amyloid precursor protein cleaving enzyme 1 (BACE1), and advanced glycation end-product (AGE) formation.

Sample	IC50 *^a^* (μg/mL)			
	AChE	BChE	BACE1	AGE Formation
Ext.	58.82 ± 9.13 **	445.55 ± 32.05 **	24.40 ± 2.84 ***	ND *^e^*
Hx fr.	33.66 ± 4.73 **	105.99 ± 0.69 ***	42.36 ± 3.94 **	166.03 ± 7.76 ***
EA fr.	80.14 ± 16.65 *	>500	64.45 ± 4.22 **	417.92 ± 14..29 ***
BuOH fr.	188.83 ± 2.44 ***	>500	ND *^e^*	ND *^e^*
Water fr.	>500	ND *^e^*	ND *^e^*	ND *^e^*
Berberine *^b^*	0.14 ± 0.01 ***	1.70 ± 0.07 **	-	-
AG *^c^*	-	-	-	104.87 ± 6.94 ***
Quercetin *^d^*	-	-	6.87 ± 0.36 **	-

Data are presented as the mean ± S.D. (n = 3); *^a^* IC_50_ calculated from the least-squares regression line of the logarithmic concentrations plotted against the residual activity; *^b^* Berberine was used as a positive control of AChE and BChE inhibition.; *^c^* AG was used as a positive control of inhibition of AGE formation; *^d^* Quercetin was used as a positive control of BACE1 inhibition; *^e^* ND was not detectable; * indicates a significant difference from control; * *p* < 0.05, ** *p* < 0.005, *** *p* < 0.001

**Table 3:** IC_50_ of the compounds **1**–**10** for acetylcholinesterase (AChE), butyrylcholinesterase (BChE), β-site amyloid precursor protein cleaving enzyme 1 (BACE1), and advanced glycation end-product (AGE) formation.

Compound	IC50 *^a^* (μM)			
	AChE	BChE	BACE1	AGEs Formation
**1**	174.83 ± 10.71 **	450.47 ± 8.82 ***	192.41 ± 7.32 ***	292.37 ± 2.28 **
**2**	147.00 ± 13.33 **	441.53 ± 10.58 **	114.63 ± 21.62 *	437.86 ± 23.94 **
**3**	187.20 ± 20.12 *	230.18 ± 5.97 **	9.29 ± 1.92 **	88.40 ± 3.28 **
**4**	27.05 ± 1.49 **	>500	>200	529.79 ± 15.53 **
**5**	19.06 ± 3.58 *	459.02 ± 13.11 **	>200	355.03 ± 12.00 **
**6**	87.19 ± 6.56 **	>500	25.89 ± 2.11 **	>1000
**7**	96.38 ± 17.23 **	>500	178.43 ± 12.15 ***	178.43 ± 12.15 ***
**8**	44.31 ± 12.20 *	>500	19.82 ± 3.05 *	522.42 ± 10.11 **
**9**	235.70 ± 21.17 **	50.43 ± 1.61 ***	>200	5.43 ± 0.11 ***
**10**	>500	>500	ND *^e^*	ND *^e^*
Berberine *^b^*	0.42 ± 0.03 *	5.05 ± 0.21 **	-	-
AG *^c^*	-	-	-	762.05 ± 69.10 ***
Quercetin *^d^*	-	-	22.75 ± 1.20 ***	-

Data are presented as the mean ± S.D. (n = 3); *^a^* IC_50_ calculated from the least-squares regression line of the logarithmic concentrations plotted against the residual activity; *^b^* Berberine was used as a positive control of AChE and BChE inhibition.; *^c^* AG was used as a positive control of inhibition of AGE formation; *^d^* Quercetin was used as a positive control of BACE1 inhibition; *^e^* ND was not detectable; * indicates a significant difference from control; * *p* < 0.05, ** *p* < 0.005, *** *p* < 0.001
